# Supplementary material for: Prevalence, knowledge and practices on prevention and management of overweight and obesity among adults in Dodoma City, Tanzania
Source: PLoS One. 2024 Jan 30;19(1):e0297665. doi: 10.1371/journal.pone.0297665 (PMC10826932; doi:10.1371/journal.pone.0297665)
Supplement: Attachment — Submitted filename: Editors Comments.rtf [file pone.0297665.s002.rtf]

Editors Comments 
Comments 	Responses 	Location in the text 	
 PLOS requires an ORCID iD for the corresponding author in Editorial Manager on papers submitted after December 6th, 2016. Please ensure that you have an ORCID iD and that it is validated in Editorial Manager. To do this, go to 'Update my Information' (in the upper left-hand corner of the main menu), and click on the Fetch/Validate link next to the ORCID field. This will take you to the ORCID site and allow you to create a new iD or authenticate a pre-existing iD in Editorial Manager. Please see the following video for instructions on linking an ORCID iD to your Editorial Manager account: https://www.youtube.com/watch?v=_xcclfuvtxQ	My updated ORCID is 

0000-0003-0646-2313		
We note that the grant information you provided in the 'Funding Information' and 'Financial Disclosure' sections do not match.
When you resubmit, please ensure that you provide the updated Financial Disclosure Statement in the Author comments box or in the Cover Letter and we will update on your behalf.
	The study is funded by the SUARIS-one project from Sokoine University of Agriculture in Morogoro, Tanzania with an award number   DPRTC/R/126/COA/5/202.	Pg.  19

Also, it is indicated in the cover letter too	
We note that you have indicated that data from this study are available upon request. PLOS only allows data to be available upon request if there are legal or ethical restrictions on sharing data publicly (http://journals.plos.org/plosone/s/data-availability#loc-unacceptable-data-access-restrictions).
In line with our goal of ensuring long-term data availability to all interested researchers, PLOS' Data Policy also states that authors cannot be the sole named individuals responsible for ensuring data access (http://journals.plos.org/plosone/s/data-availability#loc-acceptable-data-sharing-methods).
Please also note that PLOS journals will not consider manuscripts for which the authors will not share data because of personal interests, such as patents or potential future publications	Data has been shared as Supporting information file in an excel format 	19	
Before we proceed with your manuscript, please address the following prompts			
a) If there are ethical or legal restrictions on sharing a de-identified data set, please explain them in detail (e.g., data contain potentially identifying or sensitive patient information) and who has imposed them (e.g., a Research Ethics Committee or Institutional Review Board, etc.). Please provide non-author contact information (phone/email/hyperlink) for a data access committee, ethics committee, or other institutional body to which data requests may be sent. If applicable, please also provide any necessary information which interested researchers would need when requesting access to data in order to obtain the minimal data set for your study.	Data has been shared as Supporting information file in an excel format	19	
b) If your minimal data were obtained from a third party (i.e., data not owned or collected by the authors), please explain how others can access or request the specific datasets related to your research. Confirm that others would be able to access or request these data in the same manner as the authors. Please also confirm that the authors did not have any special access or request privileges that others would not have.	No restriction on data sharing hence, has been shared in an excel format	19	
c) If there are no restrictions, and your minimal data were not obtained from a third party, please upload the minimal anonymized data set necessary to replicate your study findings to a stable, public repository and provide us with the relevant URLs, DOIs, or accession numbers. Please see http://www.bmj.com/content/340/bmj.c181.long for guidelines on how to de-identify and prepare clinical data for publication. For a list of recommended repositories, please see https://journals.plos.org/plosone/s/recommended-repositories. You also have the option of uploading the data as Supporting Information files, but we would recommend depositing data directly to a data repository if possible	Data has been shared as Supporting information file in an excel form 	19
