# Supplementary material for: Prevalence, knowledge and practices on prevention and management of overweight and obesity among adults in Dodoma City, Tanzania
Source: PLoS One. 2024 Jan 30;19(1):e0297665. doi: 10.1371/journal.pone.0297665 (PMC10826932; doi:10.1371/journal.pone.0297665)
Supplement: Attachment — Submitted filename: Review.docx [file pone.0297665.s003.docx]

**Overall comments:**

The English is still not at an adequate level and should be revised again. The manuscript is too long and can be shortened easily. Reviewer comments should be addressed in the manuscript, not just in the answers to the comments, several requests have not been addressed. The sample size estimation can easily be fixed to address the previous reviewer’s comment (see below for suggestion). Line numbers would have allowed me to comment on specific words/sentences and suggest improvements.

**Specific comments:**

**Abstract:**

The abstract does not reflect the paper and should be revised to include more specific info such as the fact the study was done in Tanzania, that the aim was to inform interventions and regression was done to see which factors were associated with overweight/obesity.

**Introduction:**

Too long. Paragraphs 1 and 2 can be merged and summarized. You need to answer the following: Why is it important that Dodoma data is known? Why would your results be different from studies done in Dar? You need to justify that. For example with the fact that Dodoma is an example of a fast growing economy, fast growing population and looked at as a model city because of its administrative importance for the country. An intervention here may be easier to pilot because of the physical presence of the MoH there.

I am missing the cultural aspect introduced in the paper that is mentioned in the discussion on the fact that in Tanzania, as in many African countries, bigger body sizes are aspired to, especially for women and babies, and are mistaken for a sign of health and having achieved a good life. For men, being overweight is synonymous with a good marriage and comfortable life. This makes it hard to change perceptions.

**Materials and Methods:**

Overall: The methods section has to be revised thoroughly. Comments from a previous review were not all addressed. Smoking is still in there even though it is not used in this study. Selection criteria are not clear. Analysis is not clear. More data collected than used in the regression.

I suggest you reference well-established methods and guidelines instead of explaining how to measure height in detail. To improve your methods section, please look at your reference 27 (Khamis et al. ) and at the following paper: Gibson Benard Kagaruki, Michael J Mahande, Godfather D Kimaro, Esther S Ngadaya, Mary T Mayige, Judith Msovela, Katharina Kreppel, Sayoki G Mfinanga & Bassirou Bonfoh (2022) Knowledge and attitudes towards type 2 diabetes and prevention strategies among regular street food consumers: A cross sectional study in Dar es Salaam, Tanzania, International Journal of Health Promotion and Education, DOI: 10.1080/14635240.2022.2104742

Specifically:

The study area part should include important information such as common livelihood of the urban population in Dodoma, e.g. business. Due to the relatively recent relocation of many governmental and non-governmental organisations to the capital from Dar es salaam, population and economic growth increased in the last years. Mention this.

Sample size: see Kagaruki et al (cited below) regarding your sample size calculation, as the previous reviewer was right and hypertension prevalence is not a good replacement. Recalculate your sample size with Kagaruki’s method (your sample size should be enough) and explanation.

Please explain the abbreviation DR-NCD.

If the response rate expected is 95% you don’t need to mention that you expect a 5% non-response rate!

Training of enumerators:

Info is missing: how many? Can they speak Swahili? How big is each team? One team or more? Explain Morogoro context like: “Administration of questionnaires and dietary recall in the official language Kiswahili, was piloted using 20 volunteers in the city of Morogoro situated in another region of Tanzania, but with similar characteristics.”

State your selection criteria! Adult 18+ years, male or female, not pregnant, not suffering from a known disease, etc.

You cannot say : “requested to sign” instead you obtained informed written consent and all participants were assured they can leave the study at any time without consequences.

ID given to link questionnaire and recall data with anthropometric measurements.

Were interviews audio recorded? Transcribed? Translated? Who did quality control?

DDS: table 1 seems superfluous. Simply list the 16 food groups and reference the FAO.

The way you write it: “respondents’ views on the causes, consequences, and risk factors…” it sounds like a KAP study looking at attitudes as well as knowledge and practices. Views are attitudes, not knowledge. Adjust please. Did you do a KAP study or did you ask for knowledge and practices only?

Data analysis:

Look at other papers to improve your data analysis section. Which variables were independent? Binomial distribution etc. Which did you use in the analysis. Why did you not use all? That needs to be justified.

This is important, because you did not use all the factors you assessed! How did you include marital status? You should only have living with a partner or not living with a partner, because from a health perspective you can argue that there’s no difference between married or co-habiting.

You adjusted for age, so you have to say here what variables you used, how you combined them etc.

**Results:**

Demographics: education is seen as important when considering knowledge, so the text should reflect how many have primary, secondary or more.

Monthly income in USD please

Labour selling – revise term (day labourer)

Why is the average income only for 193 participants? What do the other 16 live off?

Table 3 only shows underweight to be significantly higher in males – there’s no significant difference for the others. Explain?

Revise this sentence and split it up, it makes no sense:

“The results show that the mean DDS for the study population was 4.7 food groups where the

most consumed food groups in 24 hours were oil and fats which were consumed by all cereals

265(84.8%), Vitamin A rich vegetables and tubers 208 (66.5%) followed by spices, condiments

and beverages (185(59.1%) of which salt and soft drinks were highly consumed in this group”

It would be interesting to know if more females or males care about their health and what the different numbers are regarding exercise (more males?).

**Discussion:**

You also cannot simply mention location names without saying in which country they are. The Monduli study was on a specific population (pastoralist) and in a rural area and cannot be compared to your study without mentioning that!

The section on limitations should include the lack of questions about body image (fat is healthy), food availability and beliefs of what is a good diet.
