# Supplementary material for: Prevalence, knowledge and practices on prevention and management of overweight and obesity among adults in Dodoma City, Tanzania
Source: PLoS One. 2024 Jan 30;19(1):e0297665. doi: 10.1371/journal.pone.0297665 (PMC10826932; doi:10.1371/journal.pone.0297665)
Supplement: Attachment — Submitted filename: Reviewers comments and aurthors responses.docx [file pone.0297665.s004.docx]

**Overall comments:**

**Comment**: The English is still not at an adequate level and should be revised again. The manuscript is too long and can be shortened easily.

**Response**: The English has been improved and poof read by an English native professor as well as shortened as suggested

**Commen**t: Reviewer comments should be addressed in the manuscript, not just in the answers to the comments; several requests have not been addressed

**Response**: responses have been incorporated in the manuscript as suggested

**Comment**: The sample size estimation can easily be fixed to address the previous reviewer’s comment (see below for suggestion)

**Response**: This has been well explained as the study is part of a large study on NCDS

**Comment**: Line numbers would have allowed me to comment on specific words/sentences and suggest improvements.

**Response**: Line numbers have been used

**Specific comments:**

**Abstract:**

The abstract does not reflect the paper and should be revised to include more specific info such as the fact the study was done in Tanzania, that the aim was to inform interventions and regression was done to see which factors were associated with overweight/obesity.

**Response:** This has been addressed accordingly **(Line 27-36)**

**Introduction**

**Comment**: Too long. Paragraphs 1 and 2 can be merged and summarized .

**Response**: The introduction has been summarized to be short as suggested **(Line 42-51)**

**Comment**: You need to answer the following: Why is it important that Dodoma data is known?

**Response:** This has been addressed in the description of the study sub-section of the methodology (**Line 89-95**)

**Comment**: Why would your results be different from studies done in Dar? You need to justify that. For example with the fact that Dodoma is an example of a fast growing economy, fast growing population and looked at as a model city because of its administrative importance for the country. An intervention here may be easier to pilot because of the physical presence of the MoH there.

**Response**: Has been addressed as suggested **(Line 92-95**)

**Materials and Methods:**

**Comment**: Overall: The methods section has to be revised thoroughly. Comments from a previous review were not all addressed. Smoking is still in there even though it is not used in this study.

**Response:** Smoking is no longer found in the manuscript as it was removed in the second version of the manuscript

**Comment:** Selection criteria are not clear. Analysis is not clear. More data collected than used in the regression.

**Response**: The analysis used many variables but they were not significant hence were removed during analysis as stated in the “note raw’ **(Line 199-202 and 256**)

**Comment:** I suggest you reference well-established methods and guidelines instead of explaining how to measure height in detail. To improve your methods section, please look at your reference 27 (Khamis et al. ) and at the following paper: Gibson Benard Kagaruki, Michael J Mahande, Godfather D Kimaro, Esther S Ngadaya, Mary T Mayige, Judith Msovela, Katharina Kreppel, Sayoki G Mfinanga & Bassirou Bonfoh (2022) Knowledge and attitudes towards type 2 diabetes and prevention strategies among regular street food consumers: A cross sectional study in Dar es Salaam, Tanzania, International Journal of Health Promotion and Education, DOI: 10.1080/14635240.2022.2104742

**Response**: The section has been improved as suggested (**Line 153-160**)

**Comment** : The study area part should include important information such as common livelihood of the urban population in Dodoma, e.g. business. Due to the relatively recent relocation of many governmental and non-governmental organisations to the capital from Dar es salaam, population and economic growth increased in the last years. Mention this.

**Response**: Addressed as suggested (**Line 93-95**)

**Comment**: Sample size: see Kagaruki et al (cited below) regarding your sample size calculation, as the previous reviewer was right and hypertension prevalence is not a good replacement. Recalculate your sample size with Kagaruki’s method (your sample size should be enough) and explanation.

**Response**: This was addressed in the first round as the study is part of the large study about NCDs **(Line 124-128**)

**Comment**: If the response rate expected is 95% you don’t need to mention that you expect a 5% non-response rate!

**Response:** Has been addressed as suggested (**Line 125**)

**Training of enumerators:**

**Comment**: Info is missing: how many? Can they speak Swahili? How big is each team? One team or more? Explain Morogoro context like: “Administration of questionnaires and dietary recall in the official language Kiswahili, was piloted using 20 volunteers in the city of Morogoro situated in another region of Tanzania, but with similar characteristics.”

**Response**: This has been addressed as suggested **(Line 131-138)**

**Comment**: State your selection criteria! Adult 18+ years, male or female, not pregnant, not suffering from a known disease, etc.

**Response**: The selection criteria was addressed as suggested (**Line 96-100)**

**Comment**: You cannot say: “requested to sign” instead you obtained informed written consent and all participants were assured they can leave the study at any time without consequences.

**Response**: Corrected in ethical consideration as suggested and the ID given to link questionnaire and recall data with anthropometric measurements. (**Line 140-143**)

**Commen**t: DDS: table 1 seems superfluous. Simply list the 16 food groups and reference the FAO.

**Response**: This has been addressed as suggested **(Line 171-175**)

**Comment**: The way you write it: “respondents’ views on the causes, consequences, and risk factors…” it sounds like a KAP study looking at attitudes as well as knowledge and practices. Views are attitudes, not knowledge. Adjust please. Did you do a KAP study or did you ask for knowledge and practices only?

**Response**: We asked for knowledge and practices hence the term views was changed to knowledge (**Line 182**)

Data analysis:

**Comment**: Look at other papers to improve your data analysis section. Which variables were independent? Binomial distribution etc. Which did you use in the analysis. Why did you not use all? That needs to be justified.

**Response**: This has been stated clearly in the note bellow the table of results as well as in the data analysis section (**Line 193-205)**

**Comment**: This is important, because you did not use all the factors you assessed! How did you include marital status? You should only have living with a partner or not living with a partner, because from a health perspective you can argue that there’s no difference between married or co-habiting.

**Response**: This has been addressed as suggested however, no significant difference was observed (**Line 256**)

**Comment**: You adjusted for age, so you have to say here what variables you used, how you combined them etc.

**Response**: Addressed as suggested (**Line 256**)

**Results:**

**Comment**” Demographics: education is seen as important when considering knowledge, so the text should reflect how many have primary, secondary or more.

**Responses:** This has been addressed accordingly **(Line 224**)

**Comment**: Monthly income in USD please

**Response**: Changes into USD as suggested (**Line 224**)

**Comment**: Labour selling – revise term (day labourer)

**Response**: This has been revised as suggested (**Line 224**)

**Comment**: Why is the average income only for 193 participants? What do the other 16 live off?

**Response:** Some participants did not agreed to mention their average income **(Line 224)**

**Comment**: Table 3 only shows underweight to be significantly higher in males – there’s no significant difference for the others. Explain?

**Response:** This has been corrected for clarity **(Line 233)**

**Comment**: Revise this sentence and split it up, it makes no sense:

“The results show that the mean DDS for the study population was 4.7 food groups where the

most consumed food groups in 24 hours were oil and fats which were consumed by all cereals

265(84.8%), Vitamin A rich vegetables and tubers 208 (66.5%) followed by spices, condiments

and beverages (185(59.1%) of which salt and soft drinks were highly consumed in this group”

**Response**: The sentence has been splitted as suggested (**Line 240-244**)

**Discussion:**

**Comment**: You also cannot simply mention location names without saying in which country they are. The Monduli study was on a specific population (pastoralist) and in a rural area and cannot be compared to your study without mentioning that!

**Response:** This has been addressed accordingly **(Line** **299-383)**

**Comment**: The section on limitations should include the lack of questions about body image (fat is healthy), food availability and beliefs of what is a good diet.

**Response**: This has been included **(Line** **384-389**)
